# Public engagement in health technology assessment in Brazil: the case of the public consultation on National Clinical Guidelines for Care in Normal Birth

**DOI:** 10.1186/s12889-021-11855-w

**Published:** 2021-10-09

**Authors:** Viviane Karoline da Silva Carvalho, Everton Nunes da Silva, Jorge Otávio Maia Barreto

**Affiliations:** 1 Campus Universitário Darcy Ribeiro, Brasília, DF CEP 70910-900 Brazil; 2 Oswaldo Cruz Foundation, Brasília, DF Brazil

**Keywords:** Social participation, Public opinion, Public consultation, Public engagement, Health technology assessment (HTA), Practice guideline, Guideline adherence, Natural childbirth, Public health policy, Analytical methods

## Abstract

**Background:**

There is a growing body of literature that recognizes the importance of public engagement in health technology assessment. However, there is still uncertainty regarding how the results should be recorded, analyzed, and used by decision makers.

**Objective:**

Synthesize the contributions of the Brazilian public (women, health professionals, managers, educational institutions, and companies) about the implementation of the National Clinical Guidelines for Care in Normal Birth from the public consultation carried out in Brazil.

**Method:**

IRaMuTeQ software was used to organize and summarize the corpus based on three types of analysis: descriptive statistics; descending hierarchical classification; and specificities analysis. The public consultation was conducted in 2016 by the National Committee for Health Technology Incorporation (CONITEC) in the Brazilian public health system as part of the guideline development process.

**Results:**

The corpus consisted of 303 texts, separated into 1233 text segments, 1081 of which were used, corresponding to retention of 87.67%. Five classes emerged from our analyses: mandatory presence of an obstetrician during labor and delivery in hospital settings; barriers and facilitators for guideline implementation; use of evidence—based practices by health professionals; progression of labor and delivery and women’s rights; and mobilization to promote the guideline For each class, the most frequent words and sentences with the highest chi-squared scores were presented. Barriers were associated with lack of financial resources, training and professional motivation, and facilitators with training to change the practices of health professionals. Obstetric nurses emerged as an alternative for supervising normal births as well as the mandatory presence of an obstetrician during childbirth in hospital settings.

**Conclusion:**

Our findings summarize the contributions provided by the Brazilian public and shed some light on the barriers and facilitators of clinical guidelines for care in normal birth. These topics are not typically explored by quantitative studies. Including this information in the decision-making process would not only increase public engagement, but provide greater evidence for implementing the clinical guidelines nationwide.

## Background

There is a growing body of literature that recognizes the importance of public engagement in health technology assessment (HTA) [1, 2], whereby communities are involved in decision-making as well as the planning, design, governance and delivery of services [3]. Public engagement, such as patient-citizens [4], for example, can improve accountability, transparency, and social inclusion in addition to providing a real-world understanding of the benefits and adverse effects of using technology to manage the process [5–7]. Public involvement, such as any member of society, can ensure the participation of individuals without personal interests, a broader representation of society and add more impartiality in decision-making in HTA [8]. It is important to highlight that the public and patient interests not always will be aligned [9], because patients could have personal interests related with the technology. However, there is still uncertainty regarding how the results should be recorded, analyzed, and used by decision makers [9–14], as well as about the impact of public, patient [12] or stakeholders involvement at HTA process [15]. According to a survey conducted by HTA agencies worldwide, barriers to involving the public in HTA processes include the potential tension between social and scientific considerations, lack of expertise in qualitative research, the mismatch between the demand for timely HTA and the time required to conduct high-quality public engagement, and the decision around who to engage in order to avoid potential biases or conflicts of interest [16].

Public consultation is frequently used by HTA agencies, whereby members of the public provide feedback on a specific technology under consideration [16]. Recently, a step-by-step approach was proposed to summarize public contributions in a systematic, transparent, reproductive, objective, and timely manner [17]. This method combines a case study based on grounded theory with the use of IRaMuTeQ software. IRaMuTeQ is used to organize, code and group words by similarity. Pearson’s chi-squared test was used at a 5% significance level to assess whether the frequency of a word was statistically associated with another [18]. It is important to apply this approach to technologies other than drug coverage decisions [17], such as clinical practice guidelines.

The Ministry of Health proposed new recommendations for care during normal birth, aimed at promoting improvements in procedures and outcomes and standardizing the most common practices in assisting normal birth. Based on the best available evidence and other international guidelines for care during normal childbirth, and using the ADAPTE methodology [19], the Brazilian National Clinical Guidelines for Care in Normal Birth reflected the consensus reached by several technical departments of the Ministry of Health, and a group of experts, professional associations (physicians and nurses), and social movements [20]. The guidelines were submitted to the National Committee for Health Technology Incorporation (CONITEC) in the public health system in December 2015. A favorable preliminary report was made available on the CONITEC website for public consultation from January to February 2016, in order to gather contributions from the public before the final decision [21].

The aim of this study is to synthesize the contributions of the Brazilian public (women, health professionals, managers, educational institutions, and companies) who participated in the consultation regarding the Brazilian National Clinical Guidelines for Care in Normal Birth, carried out by CONITEC in 2016. As proposed by Carvalho et al., 2019 [17], we used four questions that help us to summarize our main findings. The first question is about whether public categories shared some point of views on the guidelines under consultation. The second question seeks to identify whether there is public support to implement or not the guidelines under consideration. The third question refers to the main arguments (against or for) used by the public to support a decision of implementing or not the guidelines under consideration. The fourth question identifies the main issues raised by the members who participated in the consultation, stratifying by public categories. The summary provided could help both CONITEC and other HTA agencies or governments worldwide that aim to implement clinical guidelines for care in normal birth.

## Method

### Study design

A qualitative approach was used [22] to identify and synthesize the main contributions of the public consultation on implementing Brazilian National Clinical Guidelines for Care in Normal Birth. An exploratory case study was carried out to qualify the composition of the corpus, which contained all the contributions from the public consultation studied. IRaMuTeQ software was used for data processing, coding, separating and organizing information [23]. The program allows users to access text segments quickly and provides a transparent, systematic and reproducible system for processing qualitative data. IRaMuTeQ is free software that uses Python programming language and R software to statistically analyze a group of different texts combined into a corpus [24].

Based on the methodology proposed by Carvalho et al., 2019 [17], content analysis was performed using IRaMuTeQ software for data mining and to organize the corpus based on three types of analyses: descriptive statistics, descending hierarchical classification (DHC); and specificities analysis [25]. Descriptive statistics identify aspects such as the number of words, average frequency and number of hapaxes (words that only occur once) and the level of retention (percentage of text segments retained in DHC) [24, 25]. These indicators are good candidates for a measure of reproducibility of the results [17]. Descending hierarchical classification (DHC) categorizes text segments as a function of their respective vocabularies and separates them based on the frequency with which words and classes occur as well as their chi-squared scores [17, 24, 25]. Finally, specificities analysis allows text from databases to be associated with variables of interest, which in our case are the key concepts related to HTA [17, 24, 25]. These last two analyses use the chi-squared test (χ^2^) to verify whether there is correlation between any variable and words. The score of χ^2^ indicates how strong is the association between words and classes [21]. The use of chi-squared test is a default configuration on IRaMuTeQ and we decided for not change it [26].

### Data processing and analysis

Data were analyzed in four stages: First, the corpus was skim read to determine whether an alternative form of prior analysis was necessary, such as grouping contributions according to the discourse categories. We opted to work with the classification that contributors themselves declared on the public consultation form, which included nine categories: i) company; family member, friend or caregiver; iii) patient groups, associations and organizations; iv) educational institutions; v) stakeholders; vi) others; vii) patients; viii) health professionals; and ix) medical societies.

In the second stage, the corpus was prepared according to the specificities of IRaMuTeQ software, including correcting typing and punctuation mistakes, standardizing acronyms and combining compound words by adding ‘underscores’ (for example medical_obstetrician).

In the third stage, three types of analysis were performed using the IRaMuTeQ program: descriptive statistics, descending hierarchical classification (DHC) and specificities analysis (specificities and factorial correspondence analysis – FCA). For specificities analysis, 15 words related to the key concepts of HTA were selected. These were defined based on the list of the most frequently cited words in the public consultation.

In the final stage, the results were systematized and interpreted. Each class was interpreted based on the most frequent words generated using the chi-squared test. The synthesis of our main results followed the structure proposed by Carvalho et al., 2019 [17].

### Data set

The data analyzed were from the public consultation [21] about implementing National Clinical Guidelines for Care in Normal Birth [27], conducted in 2016 on the website of the National Committee for Health Technology Incorporation (CONITEC). There were a total of 396 contributions from different Brazilian states. Members of the public were asked to indicate what information they would change or include in the text; describe what would hinder implementation of the guideline, according to their own reality; explain what would facilitate its implementation, also based on their own reality; and provide additional comments on any other aspects. The fields ‘how have you contributed’ and ‘what do you think of the proposed protocol or guideline’ were used as variables in the analysis.

According to the recommendation report made available after the public consultation [27], contributions were distributed as follows: 66 from women; 24 from family members, friends or caregivers; 233 from health professionals; 63 from stakeholders; and 10 from legal entities, including companies [2], educational institutions [1], medical societies [3], patient groups/associations/organizations [2] and others [2]. Based on CONITEC’s report [28], most contributions (84%) were from women and 79% of contributors considered the guidelines good or very good, 7% fair and 14% inadequate of highly inadequate.

Researchers in the present study adopted non-participant observation, since contributions to the public consultation were collected by CONITEC. Despite the large volume of contributions, 93 were excluded from analysis because the text consisted solely of a “yes” or “no” answer. This was done to improve the quality of the corpus and the analysis itself, since these words are not related to other text segments.

### Ethical aspects

We used secondary data provided by CONITEC, which are publicly available on the internet. On this basis, there is no need for ethical approval according to the Resolution 510/16 of the Brazilian National Health Council [29].

## Results

### Characteristics of the corpus – descriptive statistics

We identified 303 texts, divided into 1233 text segments (TS), of which 1081 were used, corresponding to 87.67% TS retention (Table 1).

**Table 1:** Characterization of the corpus

Corpus	Total
Number of text^1^	303
Number of TS^2^	1233
Number of occurrences^3^	34,185
Number of word forms^4^	4248
Number of Lemmata^5^	2979
Number of active forms^6^	2788
Number of supplementary forms^7^	178
Number of Hapaxes^8^	1272 (3.72%)
TS classification^9^	1081 (87.67%)

LEGEND: 1 number of texts in the public consultation; 2 number of text segment considered by IRaMuTeQ; 3 number of words in the corpus; 4 number of word forms in the corpus. 5 number of types by headwords; 6 the main words in the corpus; 7 number of words identified as supplementary form in the corpus; 8 words used just once in the corpus; 9 a measure of text retention (TS identified by IRaMuTeQ)

Source: Elaborated by the authors

Due to the heterogeneity of text size, a TS size of 30 occurrences was adopted to guarantee greater TS retention. In order to ensure that DHC did not provide a partial classification, minimal retention between 70 and 75% was adopted [26, 30]. There were a total of 33,185 word occurrences, with 4248 different word forms and 1.272 words (3.72% of the total occurrences) that occurred only once.

### Descending hierarchical classification

**Fig. 1:**
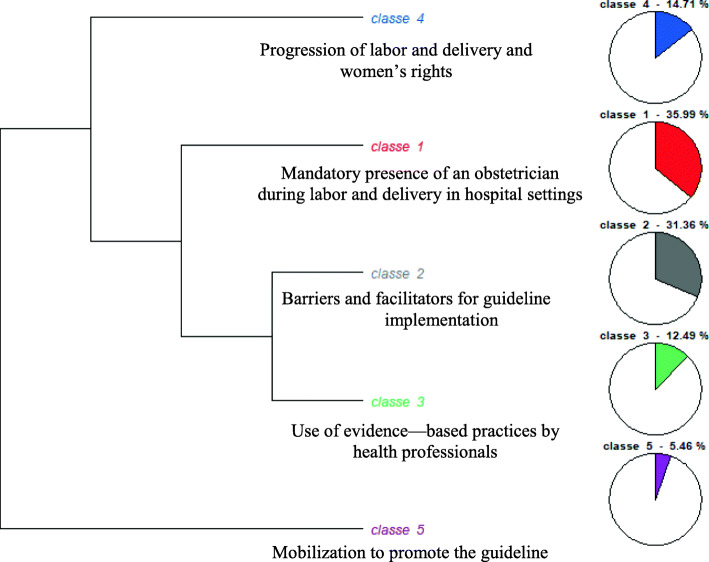
Main classes and subclasses resulting from DHC of the corpus. LEGEND: Classe 1: class 1; Classe 2: class 2; Classe 3: class 3; Classe 4: class 4 Source: compiled by the authors based on an analysis performed in IRaMuTeQ software

The content analyzed was classified by IRaMuTeQ software into five word classes (Fig. 1): class 1, with 389 TS (35.99%), class 2, with 339 TS (31.36%), class 3, with 135 TS (12.49%), class 4, with 159 TS (14.71%) and class 5, containing 59 TS (5.46%). The five classes were divided into three branches with four sub-branches: subcorpus A (class 4), subcorpus B (class 1), subcorpus C (classes 2 and 3), and subcorpus D (class 5). The main topic (subject) of each word class was identified by reading the corpus and extracting the most significant excerpts for each class (Fig. 1).

Based on analyses performed in IRaMuTeQ software, a table was compiled listing the main words, sentences, type of contribution and opinion on the guidelines for each word class (Table 2), generated using the chi-squared test (χ^2^). To extract the excerpts showed in Table 2, we first selected the typical text segment from each class. After that, we opted for displaying 50 TS and selected the excerpts with higher absolute score. Absolute score is provided by the IRaMuTeQ, considering the sum of the χ^2^ values from all words within a class.

**Table 2:** Main words, type of contribution, opinion and excerpts per class - classes 1 to 5

CLASS 1: 389 ST (35.99%) – Mandatory presence of an obstetrician during childbirth in hospital settings								
**Main Words**	**TS in the class**	**X**^**2**^	**Contributor and opinion**	**Illustrative excerpt 1 - Absolute score** **297.86**	**Illustrative excerpt 2 - Absolute score** **269.35**	**Illustrative excerpt 3 - Absolute score** **254.81**	**Illustrative excerpt 4 - Absolute score** **222.17**	**Illustrative excerpt 5 - Absolute score** **215.72**
complication	42	97.67	family member, caregiver, health_professional highly_inadequate inadequate fair	“childbirth should be performed by an obstetrician in a hospital setting to ensure the safety of mother and baby. Barriers for implementation are complications arising with no doctor to take responsibility”	“an obstetrician should always be present during childbirth. Barriers to implementation are that even uncomplicated births should always take place in a hospital setting with a team that is qualified to deal with any complication that might arise”	“obstetric nurses and midwives cannot take responsibility for complications during childbirth and there should always be an obstetrician present responsible for monitoring labor because complications are usually unexpected and require immediate medical attention”	“but suggesting that these people are more qualified than doctors or that young mothers who could give birth in a properly equipped setting should be subjected to risks and complications that cannot be dealt with at home by a legally accountable professional is absurd. Who will take legal responsibility for the consequences to mother and child if the necessary measures are not taken in the event of a complication?”	“because only doctors have the necessary technical training to deal with possible complications that might arise even in normal or uncomplicated births”
medical_obstetrician	59	70.24						
no	134	52.55						
mother	31	81.58						
childbirth	107	53.23						
see	24	85.71						
risck	33	73.33						
hospital_setting	21	87.5						
stay	17	94.44						
study	21	80.77						
possible	22	78.57						
even	35	66.04						
pediatrician	13	92.86						
complications	14	87.5						
patient	37	61.67						
**Class 2: 339 ST (31.36%) – barriers and facilitators for guideline implementation**								
**Main Words**	**TS in the class**	**X**^**2**^	**Contributor and opinion**	**Illustrative excerpt 1 - Absolute score** **1103.04**	**Illustrative excerpt 2 - Absolute score** **1082.77**	**Illustrative excerpt 3 - Absolute score** **1050.61**	**Illustrative excerpt 4 - Absolute score** **1043.00**	**Illustrative excerpt 5 - Absolute score** **1027.64**
implementation	264	323.74	Patient Very good	“barriers for implementation include resistance from the federal government and facilitators are training health professionals who provide care during childbirth especially doctors who largely follow a protocol of cesarean section deliveries and unnecessary interventions”	“barriers for implementation are the structure of the health system and the professionals involved as well as the lack of humanized care and facilitators are training and changing medical and hospital protocols”	“barriers for implementation include the poor facilities at hospitals, the lack of human resources and materials as well as an overburdened national health system. Facilitators are awareness among health professionals and greater collaboration from management”	“barriers for implementation are resistance to change on the part of health professionals lack of financial support for maternity hospitals and the municipal care model and facilitators include increasing professional training particularly obstetric nurses”	“barriers for implementation include political disinterest and facilitators are training dissemination and awareness among patients, family members and health professionals about the need for change”
barrier	193	299.63						
facilitator	160	186.21						
lack	70	95.83						
resistance	28	58.84						
professional	103	43.61						
doctor	66	39.37						
willing	16	31.6						
institution	17	30.34						
policy	13	28.8						
national_health_system	19	28.67						
care	27	26.96						
nurse	25	25.09						
training	14	23.78						
offer	15	22.97						
**Class 3: 135 ST (12.49%) – Use of evidence-based practices by health professionals**								
**Main Words**	**TS in the class**	**X**^**2**^	**Contributor and opinion**	**Illustrative excerpt 1 - Absolute score** **271.88**	**Illustrative excerpt 2 - Absolute score** **243.52**	**Illustrative excerpt 3 - Absolute score** **204.00**	**Illustrative excerpt 4 - Absolute score** **202.82**	**Illustrative excerpt 5 - Absolute score** **181.88**
scientific_evidence	22	54.9	patient other patient_groups_organizations_or_associations Very good	“caring for women during and after childbirth identifying high-risk cases and referring when needed our inclusion is vital to reduce maternal and infant mortality rates in the country as well as unnecessary cesarean deliveries and better informing the population to achieve optimum results”	“facilitators for implementation include open discussions for the community about best practices based on current scientific evidence to raise awareness in multidisciplinary teams regarding care during childbirth”	“barriers for implementation include the current culture of providing obstetric care that blatantly disregards and disrespects the latest scientific evidence in the field”	“I think it’s vital that health professionals are always up to date and trained based on scientific evidence communicating with patients and their families”	“there is no established protocol each professional does what they feel they have learned regardless of scientific evidence training human resources based on the best scientific evidence in the field”
culture	9	43.39						
based	11	43.09						
general	8	42.09						
community	5	35.2						
social	8	32.59						
population	15	32.13						
health	15	29.01						
good	11	28.91						
health_units	4	28.13						
residence	4	28.13						
extreme	4	28.13						
education	4	28.13						
empowerment	4	28.13						
women’s_health	5	27.71						
**Class 4: 159 ST (14.71%) – Progression of childbirth and women’s rights**								
**Main Words**	**TS in the class**	**X**^**2**^	**Contributor and opinion**	**Illustrative excerpt 1 - Absolute score** **983.99**	**Illustrative excerpt 2 - Absolute score** **725.68**	**Illustrative excerpt 3 - Absolute score** **717.03**	**Illustrative excerpt 4 - Absolute score** **697.51**	**Illustrative excerpt 5 - Absolute score** **604.55**
labor	58	152.28	stakeholder company Good	“item 105 page 230 if the active the stage of is not progressing the atmosphere in the delivery room should be considered and the wishes of the mother respected”	“we understand that pain relief during childbirth when needed and properly applied can favor labor progression and a healthy vaginal birth contributing to reducing unnecessary and harmful interventions such as cesarean sections”	“there is also a need to improve training given reports of professionals administering pain relief in a way that prevents the mother from moving and compromises”	“this prevents the argument that women should undergo elective cesarean sections to prevent insufficient care during childbirth if they go into labor at home and on days when healthcare teams may not be at optimal”	“we feel that every woman has the right to know and understand the physiological progression of labor as well as the risks and possible benefits of interventions during the process”
progress	21	124.19						
women	77	118.82						
progression	17	92.77						
relate	13	76.3						
lack	15	63.62						
pain	13	62.78						
want	13	62.78						
right	32	62.2						
pharmacological	10	58.53						
suspect	12	57						
pain_relief_childbirth	20	54.46						
relief	9	52.63						
diagnosis	9	52.63						
respect	20	51.92						
**Class 5: 59 ST (5.46%) – Mobilization to promote the guidelines**								
**Main Words**	**TS in the class**	**X**^**2**^	**Contributor and opinion**	**Illustrative excerpt 1 - Absolute score** **2128.05**	**Illustrative excerpt 2 - Absolute score** **2060.41**	**Illustrative excerpt 3 - Absolute score** **1429.79**	**Trecho ilustrativo 4 - Escore absoluto 1429.43**	**Illustrative excerpt 5 - Absolute score** **1171.47**
scope	22	389	other stakeholder very good good	“the research group: maternity_women_and_child_health_uff_cnpq feels that implementing these guidelines will help ensure that the labor and delivery process is an instrument for strengthening sexual and reproductive rights within the health policies of the public and private health systems”	“csm_cofen feels that implementing these guidelines will help ensure that the labor and delivery process is an instrument for strengthening sexual and reproductive rights within the health policies of the public and private health systems”	“we feel that adopting this care model will not require significant structural changes to the Brazilian health system and that similar initiatives exist within the Stork Network”	“promote the guidelines as a guiding instrument for childbirth within public and private health services and include them at state and municipal level”	“we feel that the process of compiling national guidelines based on broad debate and the involvement of different stakeholders favors a democratic society and more equitable better-quality care”
understand	26	262.59						
research group_maternity_women_and_child_health_uff_cnpq	9	157.21						
instrument	11	146.94						
initiative	11	146.94						
state	11	146.94						
described	10	142.62						
municipal	11	135.81						
implementation	15	134.69						
large	17	121.71						
chart	7	105.13						
Brazilian_health_system	6	104.51						
require	6	104.51						
protection	6	104.51						
structural	6	104.51						

Source: Elaborated by the authors

### Factorial correspondence analysis

**Fig. 2:**
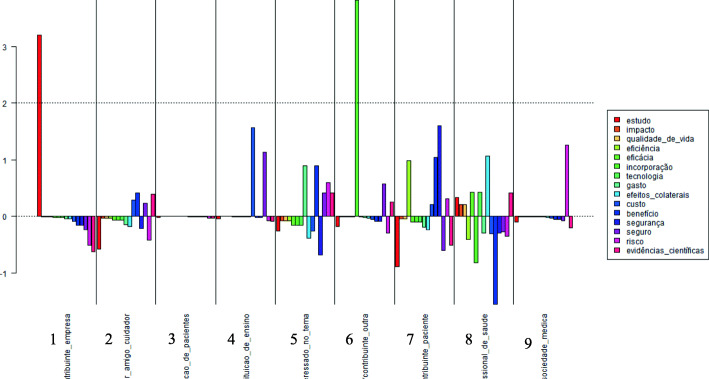
Distribution of key concepts related to health technology assessment by contributor category, Brazil, 2016**.** Source: Elaborated by the authors. LEGEND: 1 - company; 2 - family member, friend or caregiver; 3 - patient association; 4 educational institution; 5- stakeholders; 6 - other; 7 - patient; 8 - health professional; 9 - medical society.**.** Notes: From top to bottom – study, impact, quality of life, efficiency, efficacy, incorporation, technology, expenses, side effects, cost, benefit, safety, safe, risk, scientific evidence

Figure 2 shows the distribution of the 15 words related to health technology assessment (HTA) with the contributor categories provided in the public consultation. The words were selected in accordance with the HTA terms that emerged in the consultation.

The word “study” obtained the highest score under the “company” category for type of contributor (category 1), that is, the χ ^2^ calculation by IRaMuTeQ software revealed a strong statistical association between this contributor category and the word “study”, which refers to research on aspects related to childbirth, such as the benefits of giving birth at home, transfer rates to hospitals and the best position for childbirth. The words with the lowest score in this category were “scientific evidence”, “risk” and “benefit”.

The categories that showed the greatest use of key concepts related to HTA were health professionals (category 8), followed by patients (category 7) and stakeholders (category 5). The highest scoring word in the patient category was “safety”, followed by “benefit” and “efficiency”. The first two words are associated with information and guidelines on safety during labor and delivery and the benefits of natural childbirth, the presence of a doula during childbirth and the possible interventions that may occur. According to the contributions, these interventions should be properly explained to the mother to ensure she can make an informed decision about the care she wants to receive. In the public consultation, “efficiency” was related to training health professionals, particularly doctors, based on scientific evidence and communicating with patients and family members to avoid conduct based preconceptions and expert opinions.

The stakeholder category contained the words “expense”, “benefit” and “risk”. Expense and “cost” are in the same semantic field and associated with the idea that implementing the guidelines is an attempt to lower costs at health units. The word “risk” was related to the risks associated with childbirth and obtained the highest score in the medical society category.

Under health professionals (category 8), the words “side effects”, “efficacy” and “technology” were only associated with this category. “Efficacy” was related to inconclusive evidence about the use of prophylaxis for infants with chlamydial conjunctivitis, whereas “technology” and “side effects” were linked to interventions during labor. The side effects of alcohol consumption by pregnant women were also cited, as was the lack of a specific care protocol for these women.

The words with the highest score in the family member, friend or caregiver category (category 2) were “benefit” and “scientific evidence”, with the first pertaining to evidence from research that should be used to support clinical decision making.

The patient association category (category 3) showed no strong statistical association with any of the HTS words selected, whereas educational institution (category 4) was strongly associated with the words “cost” and “safe”, related to the costs of childbirth and health professionals, and labor, respectively.

According to contributors, the decision to undergo a home birth or childbirth without a doctor present is used as an alternative to lower the costs of healthcare services. The “other” category exhibited a greater statistical association with the word “incorporation”, linked to implementing the guidelines in clinical practice.

### Summary of the public consultation

A difference was observed between health professionals for the question “is there convergence/divergence of opinion between different discourse categories about the guideline under consultation?”. Some professionals argued that childbirth should be the sole domain of doctors and performed only in hospital settings, while others advocated the inclusion of obstetric nurses and midwives in normal births. In general, although the remaining categories contained different ideas, these did not characterize differences of opinion. While some stakeholders argued in favor of mobilizing to promote the guidelines, others advocated for allowing labor to progress naturally and respecting women’s right to choose. Despite being different topics, they are not opposing ideas, as occurred for health professionals.

With regard to the question “is there public support for including/excluding the guideline under consultation?”, in general there were more arguments in favor of implementing the guidelines. A portion of contributors from both categories (health professionals and family members, friends or caregivers) were more emphatic in opposing the guidelines because they felt the presence of a doctor was vital during childbirth to address any complications that might arise.

Some of the barriers cited under the question “what are the main pros/cons raised by members of the public about including/excluding the guideline under consultation?” were the resistance of health professionals, lack of teams willing to perform home births and shortage of human resources and materials. The main arguments in favor of the guidelines are related to the inclusion of obstetric nurses and midwives, respecting women’s right to choose and mobilization to promote the guidelines. Some contributors viewed the guidelines as an instrument to support professional practices and strengthen sexual and reproductive rights within healthcare policies. Facilitators mentioned were determination, training programs and professional awareness.

**Fig. 3:**
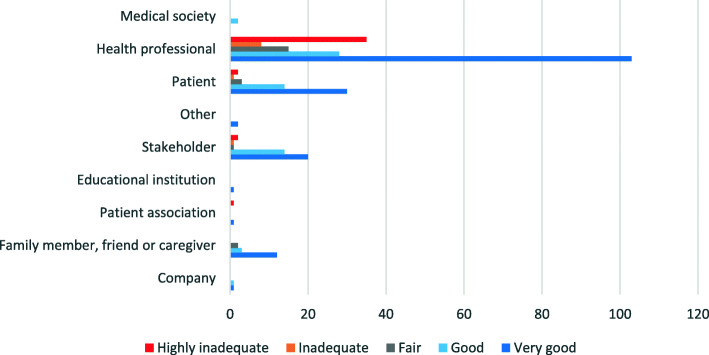
Guideline assessment by type of contributor. Source: compiled by the authors based on 303 contributions included in analyse

Finally, for the question “what are the main issues related to the opinion and type of contributor who participated in the public consultation”, only a small portion of health professionals, patient associations, stakeholders and patients viewed the guidelines as negative, with opinions of “highly inadequate”, “fair” and “inadequate” predominating. The opinion of the remaining contributors ranged from “fair” to “very good” (Fig. 3).

The main arguments in favor of the guidelines were related to introducing obstetric nurses and midwives to maternity wards and creating a law or ordinances to regulate the guidelines. The inclusion of obstetric nurses, offering fair salaries and a mandatory requirement for all nurses caring for pregnant and laboring women to hold a specialty in obstetrics were highlighted by health professionals and medical societies. Stakeholders and companies emphasized that the inclusion of obstetric nurses and midwives could contribute to respecting and guaranteeing women’s right to choose. Contributors who identified as stakeholders and others addressed issues related to the support of research groups and health committees in mobilizing to promote the guidelines.

## Discussion

In global terms, a recent systematic review found that clinical guidelines for uncomplicated birth are generally diverse, exhibit poor methodological quality and low agreement in terms of recommendations [31]. The lack of international consensus may explain the different opinions among participants in the public consultation. A key point of the consultation was the conflict between health professionals about the possibility of obstetric nurses or midwives supervising the birth. The International Confederation of Midwives (ICM) defines a “midwife” as someone with the necessary qualifications to be registered and/or legally licensed to practice midwifery, in accordance with the ICM Global Standards for Midwifery Education [32]. In the United Kingdom, most care during childbirth is provided by midwives working in partnership with doctors in vaginal births and cesarean sections to support the mother and baby [33].

Issues related to a lack of understanding regarding the rights of pregnant women were also mentioned. Women can experience different forms of obstetric violence and its causes are multifactorial, requiring the combined efforts of different health professionals to address the problem [34]. Creating an environment of care is one of the most challenging aspects and suggests that midwifery acts in defense of women within the healthcare system [35]. For example, some women associate overcoming their fears and doubts and building confidence in their ability to give birth without pharmacological pain relief to the relationship of trust with midwives [36]. In Brazil, the Ministry of Health launched the Stork Network in 2011 at the regional level to provide a care network aimed at guaranteeing women the right to reproductive planning and humanized care through pregnancy, childbirth and the postpartum period [37]. The Stork Network consists of four components that involve a series of healthcare initiatives: prenatal care; childbirth; postpartum and comprehensive child care; and logistics, sanitary transport and regulation. Its implementation in Brazilian states and municipalities is based on epidemiological criteria such as population density and infant and maternal mortality rates [37].

The key HTA concepts were little used by participants in the public consultation. Of the nine contributor categories, only three used more than four HTA-related words in their contributions, suggesting that both these terms and the importance of evidence-based decision making need to be better disseminated among public consultation participants. The need for HTA education and training strategies for patients and the public was also identified in research performed by HTA agencies that evaluated their public and patient engagement processes [38]. Additionally, it has been reported that patient organizations generally do not receive training from HTA agencies [39].

### Strengths and limitations of the study

Our study contributed by applying the method proposed by Carvalho et al. 2019 [17] to a clinical practice guideline, and proved to be effective with satisfactory results. This method was previously applied to a public consultation regarding the incorporation of a drug in the public health system. This method combined with IRaMuTeQ software was also faster than conventional content analysis, which is beneficial for HTA agencies, given the short time period they have available to conduct this type of assessment. Descriptive statistics were also used as a likely indicator of reproducibility should other researchers be interested in replicating the analysis. Reproducibility is also a vital attribute for health technology assessment. In addition, the present study progressed in such a manner that the opinions, experiences and interests of contributors to the public consultation on National Clinical Guidelines for Care in Normal Birth were systematized.

However, the study also exhibited some limitations. First, 12.33% of text segments were not used in the DHC analysis conducted in IRaMuTeQ software, obtaining retention of 87.67%. Although this loss may have influenced the results to some degree, retention was still the minimum 75% recommended by the IRaMuTeQ manual [26]. Second, the contributor categories used were those defined by CONITEC for the public consultation and some of these may have overlapped for some contributors, such as “pregnant woman” and “health professional”. However, we opted to use contributors’ self-reported categories since they are better equipped to know which categories best represent them. Third, limitations regarding the public engagement process in Brazil were also identified. Based on the dissemination strategies of the public consultation used by CONITEC (website and email lists), the participants probably had some interest in the subject or knew about the processes adopted by CONITEC. It is likely that participants attracted by the public consultation may not represent the Brazilian society, both because of the small number of contributions received and because of the limited forms of dissemination adopted by CONITEC. People who do not have access to the internet, for example, are not able to participate in the public consultation. Although these limitations are not caused by the method used, we consider that the analysis is valid to highlight aspects about this group of people involved with CONITEC process. It is important to mention that the Brazilian engagement process in HTA needs to be improved. Finally, we used a public consultation carried out in 2016, after CONITEC had made the final decision on National Clinical Guidelines for Care in Normal Birth. Although this study lost its potential to contribute to decision making at the time, it is still relevant in terms of highlighting aspects of the guidelines that should be monitored following their implementation. The information summarized here can also be used in other contexts or countries that initiate discussions regarding normal birth.

### Implications for public policies

Identifying and addressing the barriers to implementation is critical to the success of a healthcare policy or program [40]. The National Clinical Guidelines for Care in Normal Birth are a valuable tool for integrating scientific knowledge into the practices of health services and care providers in order to improve health outcomes and people’s lives. However, their implementation requires knowledge of the importance of qualitative evidence in this process [41–43]. Qualitative evidence is increasingly valued worldwide, including global academic efforts to make it more systematic, transparent and reliable [44]. The present study contributes to this field by providing a practical application of a tool to support the analysis and synthesis of colloquial qualitative evidence, as well as a set of important elements for decision makers to consider when planning the implementation of guidelines for normal birth in Brazil and similar contexts.

## Conclusions

This study systematized opinions, experiences, and interests of contributors from the public consultation on the National Clinical Guidelines for Care in Normal Birth in Brazil. Based on that, we could achieve our objective of identifying barriers and facilitators related to the clinical guidelines’ implementation. The inclusion of obstetric nurses and midwives in normal births was a point of divergence between healthcare workers. Few healthcare workers argued that childbirth should be performed by doctors only at hospital setting. We identified more arguments in favor of implementing the guidelines than in opposing, and only a small portion of contributors viewed the guidelines as negative. Some barriers identified were related to human resources and healthcare worker’s resistance to perform home births. Determination, training programs and healthcare worker awareness, as for example about the need for change, were mentioned as facilitators.

The low use of key HTA concepts by the public consultation participants was identified, what suggests a lack in knowledge or in training from the participants. To qualify the HTA social participation, the training and dissemination of information about HTA process and evidence-based decision making to public consultation participants must be improved by HTA agencies.

Finally, our study explored topics that are not typically explored by quantitative studies. This kind of information can help to improve the HTA process, increase public engagement and provide greater evidence for implementing the clinical guidelines in Brazil and other counties with similar context.

## Data Availability

The datasets analysed during the current study are available in the CONITEC repository, [http://conitec.gov.br/images/Consultas/Contribuicoes/2016/CP_CONITEC_01_2016_PCDT_Diretriz_Nacional_de_Assistência_ao_Parto_Normal.pdf].

## Abbreviations

CONITEC: National Committee for Health Technology Incorporation
DHC: Descending hierarchical classification
FCA: Factorial correspondence analysis
HTA: Health Technology Assessment
IRaMuTeQ: Interface de R pour les Analyses Multidimensionnelles de Textes et de Questionnaires)
SUS: National Health System
TS: Text segments
χ2: Chi-squared
